# MRI Image Segmentation Model with Support Vector Machine Algorithm in Diagnosis of Solitary Pulmonary Nodule

**DOI:** 10.1155/2021/9668836

**Published:** 2021-07-20

**Authors:** Bo Feng, Meihua Zhang, Hanlin Zhu, Lingang Wang, Yanli Zheng

**Affiliations:** Department of Radiology, Hangzhou Ninth People's Hospital, No. 98 Yilong Road, Yipeng Street, Qiantang District, Hangzhou 311225, Zhejiang Province, China

## Abstract

This study focused on the application value of MRI images processed by a Support Vector Machine (SVM) algorithm-based model in diagnosis of benign and malignant solitary pulmonary nodule (SPN). The SVM algorithm was constrained by a self-paced regularization item and gradient value to establish the MRI image segmentation model (SVM-L) for lung. Its performance was compared factoring into the Dice index (DI), sensitivity (SE), specificity (SP), and Mean Square Error (MSE). 28 SPN patients who underwent the parallel MRI examination were selected as research subjects and were divided into the benign group (11 patients) and malignant group (17 patients) according to different plans for diagnosis and treatment. The apparent diffusion coefficient (ADC) at different *b* values was analyzed, and the steepest slope (SS) and washout ratio (WR) values in the two groups were calculated. The result showed that the MSE, DI, SE, SP values, and operation time of the SVM-L model were (0.41 ± 0.02), (0.84 ± 0.13), (0.89 ± 0.04), (0.993 ± 0.004), and (30.69 ± 2.60)s, respectively, apparently superior to those of the other algorithms, but there were no statistic differences (*P* > 0.05) in the WR value between the two groups of patients. The SS values of the time-signal curve in the benign and malignant groups were (2.52 ± 0.69) %/s and (3.34 ± 00.41) %/s, respectively. Obviously, the SS value of the benign group was significantly lower than that of the malignant group (*P* < 0.01). The ADC value with different *b* values in the benign group was significantly lower than that of the malignant group (*P* < 0.01). It suggested that the SVM-L model significantly improved the quality of lung MRI images and increased the accuracy to differentiate benign and malignant SPN, providing reference for the diagnosis and treatment of SPN patients.

## 1. Introduction

In China, lung cancer ranks first for the incidence and mortality among malignant tumors. Worldwide, lung cancer has the highest mortality and its incidence ranks third. Moreover, the number of patients with lung cancer is constantly growing and the survival rate of lung cancer in China is much lower than that in developed countries [1]. The CT image of SPN is of high density with the *D* ≤ 3 cm, distinct or indistinct borders, and round or irregular shapes. Single round or oval nodules in the lung parenchyma are not accompanied with pulmonary atelectasis and lymphadenectasis and the like [2]. SPNs are roughly classified into benign and malignant ones, but the majority is indistinguishable [3]. The commonly used MRI imaging methods include X-ray plain film, MSCT routine scan, enhanced dynamic scan, and computed tomography perfusion (CTP). The X-ray easily misses the hidden part or small lesions, and its detection rate of SPNs in adult lungs is only 0.1%–0.2% [4]. PET or PET-CT still has the problem of missed diagnosis, which may increase the false negative rate [2]. CTP and spectral CT technology are efficient in diagnosing pulmonary nodules, but they require a high radiation dose, which limits their clinical applications [5].

Recently, MRI has been widely applied in diagnosis of a variety of diseases. It can reduce susceptibility artifact and cardiovascular beat artifact arising from the air-tissue interface when diagnosing lung diseases. MRI is characterized by multiple-layer and multiple-sequence imaging and intensified signals. It can also reflect the number of capillaries and blood perfusion parameter values of benign and malignant lung nodules.

The dynamic contrast-enhanced-magnetic resonance scan (DCE-MR) is a conventional noninvasive imaging method to observe physiological characteristics of tumor. The blood perfusion parameter values can be used as the imaging biomarkers to differentiate benign and malignant nodules. However, there is no unified standard and qualitative threshold for DCE-MR in evaluating benign and malignant SPNs [6]. SVM has achieved excellent results in the field of the medical image segmentation. Yamamoto et al. [7] used SVM algorithm to segment white matters, gray matters, and other categories of MRI images for brain; Sun et al. [8] used SVM algorithm to segment MRI images for prostate tumor. However, the SVM algorithm has rarely been applied to segment MRI images for the lung.

All in all, the SVM algorithm has already been applied to segment MRI images for multiple organs, but rarely used in lung segmentation. In the study, the SPN patients were chosen as research subjects, and the SVM-based algorithm was used to process the MRI images for the lung, to differentiate benign and malignant SPNs, expected to provide a theoretical basis for diagnosis and treatment of SPN.

## 2. Materials and Methods

### 2.1. Subjects and Grouping

28 confirmed SPN patients, who received thoracic surgery in the hospital from June 2019 to September 2020, were chosen as subjects, and all patients underwent MRI examination, including 9 male patients and 19 female patients. The age of patients ranged from 19 to 65 years and the average age was 51.04 ± 6.52 years. All the patients were divided into a benign group and malignant group according to different clinical diagnoses and treatments. The benign group was made up of 11 patients, while the malignant group had 17 patients. The inclusion criteria for the study are shown as follows: (1) The chest CT examination in the hospital was chosen to specify the solid nodules and lesions (≥1 cm, ≤3 cm) inside the lungs; (2) the lesions had uniform density and no calcification or cavitation and no satellite lesions around, with no pulmonary atelectasis and lymphadenectasis caused; and (3) all patients who got lesions after operations; their pathological results were confirmed as sarcoidosis, and they had complete image materials. Exclusion criteria: (1) the patients who failed to receive DCE-MRI scanning; (2) the artifact in the MRI image affected the observation of lesions; (3) the patients with pulmonary atelectasis and pneumonia around lesions; and (4) the patients who suffered from malignant tumors in other parts. The procedure was approved by the ethics committee of the hospital, and all the subjects who were included in the study signed the informed consent form.

### 2.2. Establishment of an SVM-Based MRI Image Segmentation Model

Through nonlinear transformation, SVM transforms the input space into a high-dimensional space, in which the optimal linear discriminant surface is calculated. The medical image data are usually linear and undividable [9]. The data in the initial space are mapped to a new space using nonlinear transformation. Provided that *ϕ*(*x*) is a mapping function, *x*_*i*_ is an m-dimensional input vector. Its kernel function is expressed as

(1) $$\begin{array}{c} K\left(x,x_{i}\right)=\phi \left(x\right)\phi \left(x_{i}\right). \end{array}$$

Under this condition, the nonlinear SVM model is expressed as

(2) $$\begin{array}{c} \min\frac{1}{2}\sum_{i=1}^{n}\sum_{i=1}^{n}\alpha_{i}\alpha_{j}y_{i}y_{j}K\left(x_{i}x_{j}\right)-\sum_{i=1}^{n}\alpha_{i},\\ \text{s}\mathrm{.t}.\;\sum_{i=1}^{n}y_{i}\alpha_{i}=0,\;0\leq \alpha_{i}\leq c. \end{array}$$

In the abovementioned equation, *c*represents the penalty function to balance interval classification and misclassification, *x*_*i*_ indicates the m-dimensional input vector, *y*_*i*_ is the label of the sample, *n* represents the number of training samples, and *α*_*i*_ indicates the slack variable.

In the MRI image for the lung, the eigenvector for each pixel is *x* and its training dataset is expressed as *A*={(*x*_*i*_, *y*_*i*_), *i*=1,2,…, *n*}, where *x*_*i*_ represents the no. I training sample, *y*_*i*_ indicates the real label of no. *i* training sample, and *n* is the number of training sample. Based on characteristics of the MRI image for the lung, the self-paced regularization item is added to the article so as to constrain the SVM model. The specific calculation method for the self-paced regularization item is shown as follows:

(3) $$\begin{array}{c} \underset{w,a,v}{\min}\sum_{i=1}^{n}v_{i}L\left[y_{i},g\left(wx_{i}+a\right)\right]+\eta {\left\|w\right\|}^{2}+f\left(v_{i},\lambda \right). \end{array}$$

In equation (3), *L*[*y*_*i*_, *g*(*wx*_*i*_+*a*)] indicates the training error of each sample, *v*_*i*_ represents the weight, *w* is the decision function parameter, *λ* indicates the instantaneous function parameter, *η* is the step length, and *a* is the penalty coefficient.

Provided that *x*_*i*_ is the sample set, *i*=1,2, ⋯, *m*, and *y*_*i*_ is the category label number of sample set, (*x*_1_, *y*_1_),…, (*x*_*i*_, *y*_*i*_) ∈ *R*^*D*^ × {−1,1}, the optimization problems for SVM are expressed as follows:

(4) $$\begin{array}{c} \min\frac{1}{2}{\left\|\omega \right\|}^{2}+C\overset{n}{\underset{i=1}{\sum }}\alpha_{i},\\ \text{subjected to }y_{i}\left(\omega \cdot x_{i}+b\right)\geq 1-\alpha_{i},\;\alpha_{i}>0,\;\left(i=1,2,\ldots ,m\right). \end{array}$$

In equation (4), *C* is the regularization parameter, *α*_*i*_ is the slack variable, *ω* is the weight vector, and *b* is the error item.

The discriminant function of SVM is shown in equation (2), in which *Ns* represents the number support vector, *β*_*i*_ is the multiplier of Lagrange and meets the condition of *β*_*i*_ > 0, *G*(*x*, *x*_*i*_) is the kernel function, and *c* is the threshold value of classification.

(5) $$\begin{array}{c} f\left(x\right)=\sum_{i=1}^{Ns}y_{i}\beta_{i}G\left(x,x_{i}\right)+c. \end{array}$$

Because the kernel functions are different, SVM algorithms vary greatly from each other. In general, the kernel function is only required to meet Mercer theorem [10]. In this article, the radial basis is chosen as the kernel function and expressed in equation (3), in which *γ* is the radial basis parameter.

(6) $$\begin{array}{c} K\left(x,x_{i}\right)=\exp\left(-\gamma {\left\|x-x_{i}\right\|}^{2}\right). \end{array}$$

If a group of training samples can be divided by the optimal discriminant surface, the expected value of the error rate of testing samples meets the following condition:

(7) $$\begin{array}{c} E\left[P\left(\text{error}\right)\right]\leq \frac{E\left(\text{SV}\right)}{n}. \end{array}$$

In equation (7), *n* represents the number of training sets, SV is the support vector, *E*[*P*(error)] indicates the expected value of the error rate of testing samples, and *E*(SV) is the expected value of the number of support vector. The correct rate discriminant weight *w*_*t*_ is expressed as follows: *w*_*t*_=1 − (*E*(*SV*)/*n*).

In MRI image segmentation for the lung, how to precisely separate the border parts of the lung and remove the adhesion between the lung and peripheral organs are the core parts of segmentation algorithm. In the MRI image, the pixel at the texture border usually has a higher gradient value. The information on the gradient value of textures is collected to enhance the expression of texture characteristics. The gray-level and gradient-level co-occurrence matrix [11] combines information on the gray level and gradient level in the image and highlights the direction of texture. The pixel in the image is taken as the center to construct a rectangle block (5 × 5) and extract the characteristics of the gray level and gradient level. The calculation method for advantages of small gradient is shown as follows:

(8) $$\begin{array}{c} \mu_{1}=\frac{\left[\sum_{i=1}^{n}\sum_{j=1}^{n}\left(H\left(i,j\right)/j^{2}\right)\right]}{\left[\sum_{i=1}^{n}\sum_{j=1}^{n}H\left(i,j\right)\right]}. \end{array}$$

The calculation method for the inverse difference moment is shown as follows:

(9) $$\begin{array}{c} \mu_{2}=-\sum_{i=1}^{n}\sum_{j=1}^{n}\frac{1}{1+{\left(i-j\right)}^{2}}P\left(i,j\right). \end{array}$$

In equations (8) and (9), *H*(*i*, *j*) represents the number of pixels in the rectangle block (5 × 5), with a pixel as the center, *i* as the gray level, and *j* as the gradient value. *P*(*i*, *j*) indicates the probability of co-occurrence of the gray level (*i*) and gradient (*j*) in the rectangle block (5 × 5) with the currently calculated pixel as the center.

**Figure 1:**
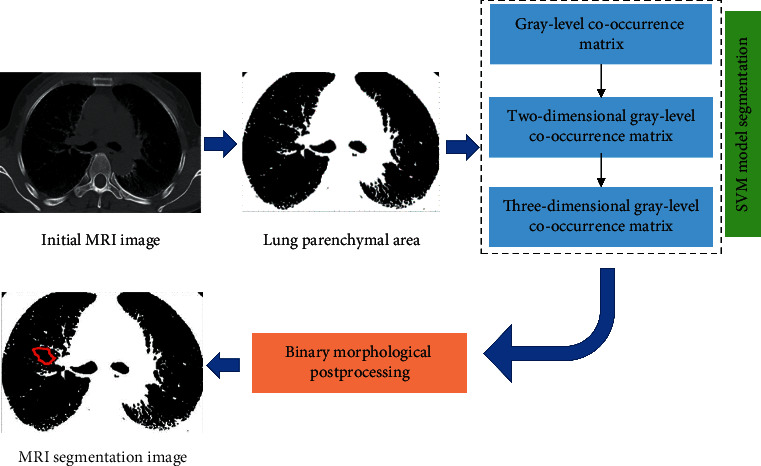
Segmentation flow for the SVM-L-based MRI image.

In this article, the MRI image segmentation method based on optimized SVM is named as SVM-L. The scanned MRI image is processed through the image processing software to obtain the lung parenchyma area, passes through co-occurrence matrix algorithms of different dimensions, gray levels, and gradient values to construct the characteristics vectors, undergoes the segmentation treatment using the VSM model, and then, experiences the posttreatment of 2D morphology to output the MRI segmentation image. The segmentation flow for the SVM-L model MRI image is shown in Figure 1.

### 2.3. Comment on Quality of the SVM-Segmented MRI Image

The segmentation results of the MRI image for the lung are analyzed in terms of Dice Index (DI), sensitivity (SE), specificity (SP), and mean square error (MSE). The Dice index is to measure whether the segmentation results and gold standard area are overlapped. The calculation equation for the Dice index is shown as follows:

(10) $$\begin{array}{c} \text{Dice}\left(A,B\right)=2\times \frac{A\cap B}{A\cup B}. \end{array}$$

In equation (10), A represents the standard value segmented by a doctor and B represents the predicted value segmented by the SVM-based model. The value range of the Dice index is [0,1]. The Dice index is increasingly rising, which means the predicted result is closer to the real value.

The sensitivity is mainly to measure and segment the proportion of the correctly detected target area, while the specificity is used to assess the ratio of automatic segmentation to the exclusion of nontarget areas. The algorithms for sensitivity and specificity are shown as follows:

(11) $$\begin{array}{c} \text{SE}=\frac{\left|A\cap B\right|}{B},\\ \text{SP}=\frac{\left|A_{1}\cap B_{1}\right|}{B_{1}}. \end{array}$$

In equation (11), *A*_1_ and *B*_1_ represent the complementary set of set *A* and *B*. The value range of SE and SP is [0,1]. The higher the value range of SE and SP is, the better the segmentation effect is.

The calculation method for MSE is shown as follows:

(12) $$\begin{array}{c} \text{MSE}\left(f,g\right)=\frac{1}{\text{MN}}{\sum_{i=1}^{M}\sum_{j=1}^{N}\left(f\left(i,j\right)-g\left(i,j\right)\right)}^{2}. \end{array}$$

In equation (12), *f* represents the MRI image segmented by a doctor, *g* represents the SVM model segmentation image, *M* × *N* represents the dimension of the segmented image, and *i* and *j* are pixel values. The smaller the MSE value is, the better the image quality is.

### 2.4. MRI Scanning Method

The SIEMEMENS 3.0 T magnetic resonance machine is used to conduct routine MRI flat scanning and DCE-MRI scanning for all patients. The routine MRI scanning: (1) the repetition time (T1) of weighted imaging of axial electrocardiogram gating spin echoes is 1800 ms, spin echo time is 2.4 ms, slice thickness is 5 mm, slice gap is 1 mm, scan field of view is 360 mm, and scanning time is 73s. (2) The repetition time (T2) of weighted imaging of fast spin echoes is 3000 ms, spin echo time is 83 ms, slice thickness is 5 mm, slice gap is 1 mm, scan field of view is 360 mm, and scanning time is 195s. (3) The thickness of the DCE-MRI-enhanced scan slice is 3.6 mm, slice gap is 1.2 mm, scan field of view is 380 mm, and flip angle is 2°. At the dose of 0.2 mmol/Kg and rate of 0.2 ml/s, the dimeglumine Gadopentetate is injected through the cubital vein. In 30 seconds after injection, the no. 30 phase of the nodule area scanning is conducted.

### 2.5. MRI Image Processing Method and Observation Indicators

The MR-scanned data are transmitted to the Siemens syngo MRD image processing workstation. The quantitative parameter values (DCE-MRI) of all data are measured by 2 physicians who have more than 3 years' experience in the radiology department, and the mean value of the 2 physicians is determined as the final data. Three regions of interest (ROIs) on the solid part of lesions with the largest sections are sketched manually, to avoid the measured scope including calcification, necrosis, cystic change, and hemorrhage area. Each ROI covers an area of 0.3cm^2^–0.5 cm^2^.

At different *b* values, the ADC values of ROI of two groups of patients are analyzed. The obtained data are used to draw a time-signal curve. The curve type is determined according to the classification standard of Schaefer. The proportion of two groups of patients in different curve types is calculated. With time as the *X*-axis and signal intensity variation as the *Y*-axis, the SS and washout ratio (WR) are calculated.

### 2.6. Statistical Method

The SPSS19.0 statistical software is used to process experimental data, and the mean value of measurement data ± the standard deviation is expressed as $\bar{x}\pm s$. The comparison of mean values of all groups is verified by *t*. The measurement data are expressed by percent (%) and tested by *χ*^2^, and *P* < 0.05 indicates the statistical significance in differences.

## 3. Results

### 3.1. Analysis on the Result of SVM-Based MRI Image Segmentation

**Figure 2:**
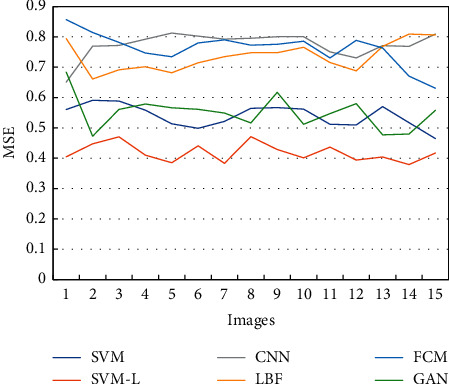
Comparison of MSE values of MRI images segmented through different algorithms.

In this article, the MSE value of the SVM-based optimized SVM-L model was compared with that of fuzzy C-means (FCM), Convolutional Neural Network (CNN), SVM, local binary fitting (LBF), and Generative Adversarial Network (GAN) algorithms. The comparison result showed that, in 15 detected MRI images, the MSE value of SVM-L models was apparently lower than that of other algorithms and the average MSE value was 0.41 ± 0.02 (Figure 2).

**Figure 3:**
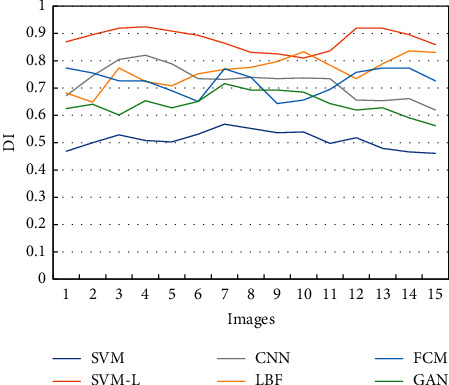
Comparison of Dice indexes of MRI images segmented through different algorithms.

The DI values of MRI images segmented through different algorithms were compared (Figure 3). The comparison result showed that the DI values of SVM-L models in 15 detected MRI images were apparently lower than those of other algorithms and the average DI value was 0.84 ± 0.13.

### 3.2. Analysis on SE and SP of SVM-Based MRI Segmentation

**Figure 4:**
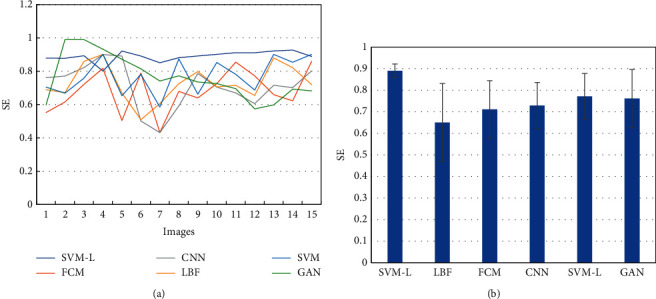
Comparison of SE of MRI images segmented through different algorithms. (a) Distribution of SE values of MRI images segmented through different algorithms; (b) comparison of average SE values of MRI images segmented through different algorithms.

When 15 MRI images were processed through different algorithms, the SE value of the SVM-based optimized SVM-L model was compared with that of other five algorithms. The result showed that the SE values had higher volatility and the SE value of SVM-L models was relatively higher than that of other algorithms. The average SE value of the SVM-L model was 0.89 ± 0.04, while the average SE value of FCM, CNN, LBF, and GAN algorithms was 0.68 ± 0.13, 0.71 ± 0.13, 0.73 ± 0.11, 0.77 ± 0.10, and 0.76 ± 0.13, respectively (Figure 4). The average SE value of the SVM-L model was obviously higher than that of other algorithms.

**Figure 5:**
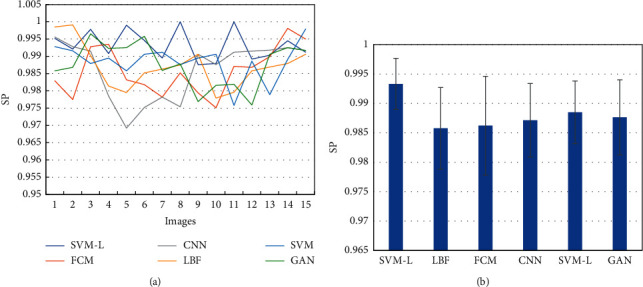
Comparison of SP of MRI images segmented through different algorithms. (a) Distribution of SP values of MRI images segmented through different algorithms; (b) comparison of average SP values of MRI images segmented through different algorithms.

When MRI images were processed through different algorithms, all the SP values had a higher volatility (Figure 5(a)). The average SE values of 15 different MRI images segmented through different algorithms were calculated and analyzed (Figure 5(b)). The average SP value of SVM-L model, FCM, CNN, LBF, SVM, and GAN algorithms was 0.993 ± 0.004, 0.986 ± 0.006, 0.986 ± 0.008, 0.987 ± 0.006, 0.988 ± 0.005, and 0.987 ± 0.006, respectively. The average SP value of the SVM-L model was apparently higher than that of other algorithms.

### 3.3. Analysis on Time of SVM-Based MRI Segmentation

**Figure 6:**
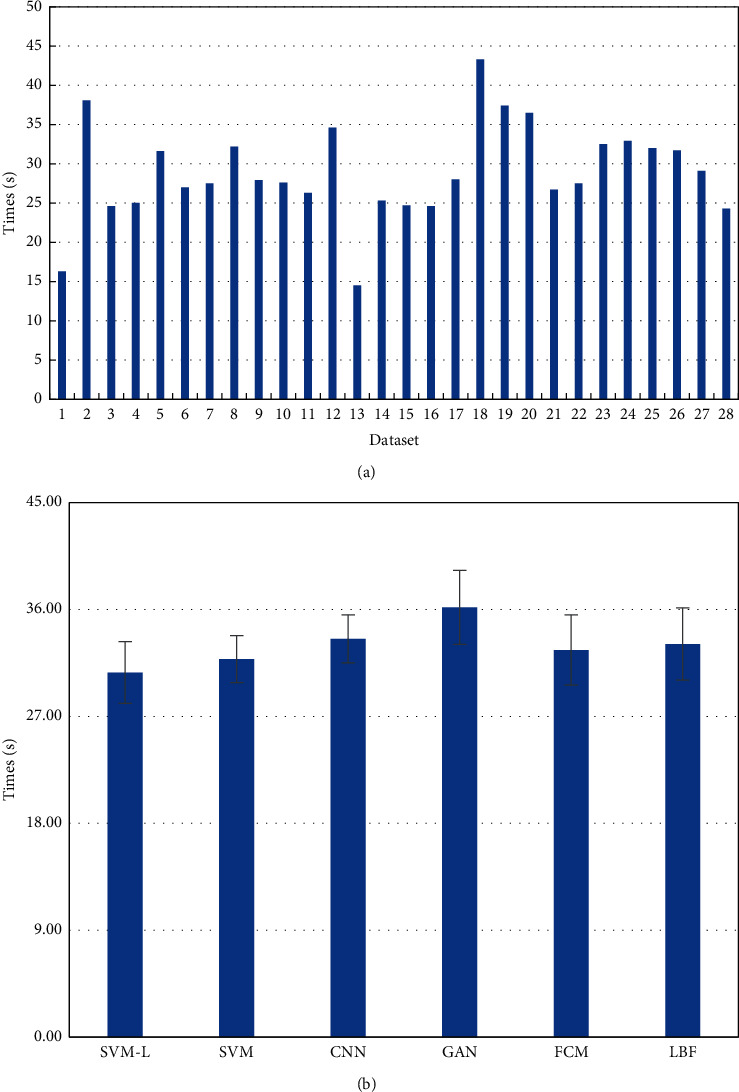
Comparison of segmentation time of MRI images segmented through different algorithms.

When 28 different MRI images were processed through the SVM-L model, the segmentation time was as shown in Figure 6(a). The segmentation time of different MRI images varied greatly from each other and ranged from 14.15 s to 43.06 s. The average segmentation time of 28 MRI images processed through different algorithms was further compared (Figure 6(b)). The average segmentation time of SVM-L model, FCM, CNN, LBF, SVM, and GAN algorithms was 30.69 ± 2.60, 36.52 ± 2.02, 38.17 ± 3.11, 37.85 ± 2.95, 35.83 ± 1.98, and 39.07 ± 3.03, respectively. The segmentation time of MRI images of the SVM-L model was evidently lower than that of other algorithms.

### 3.4. MRI Features of SPN Patients

**Figure 7:**
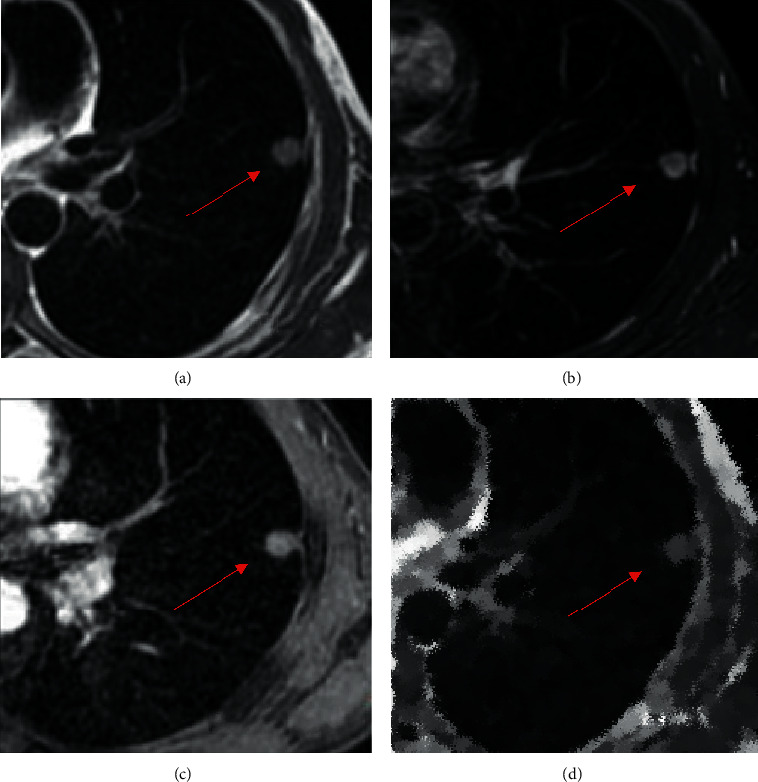
MRI features of SPN patients. (a) T1W1 image; (b) T2W1 image; (c) DW1 image; and (d) ADC image.

In this article, the MRI images of SPN patients were processed through the SVM-based optimized MVM-L model to analyze the MRI features of SPN. The lesions of SPN patients were oval in shape (where the red arrow is) and had nonuniform signals and rough borders. T1W1 showed that the lesion had equivalent signals (Figure 7(a)), T2W1 showed that the lesion had distinctly higher signals (Figure 7(b)), DW1 indicated that the lesion with rough borders had obviously high signals (Figure 7(c)), and ADC indicated that the lesion had low signals (Figure 7(d)).

### 3.5. Comparison of Time-Signal Curve Types of MRI Images of SPN Patients

**Figure 8:**
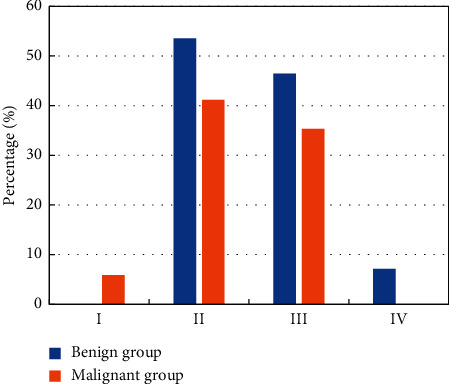
Comparison of time-signal curve types of MRI images of the two groups of SPN patients.

The statistics on time-signal curve types of MRI images of SPN patients was analyzed (Figure 8). There were no statistical differences in time-signal curve types of different MRI images of the two groups of patients in terms of the proportion of patients (*P* > 0.05).

### 3.6. Comparison of Parameters for the Time-Signal Curve of MRI Images of SPN Patients

**Figure 9:**
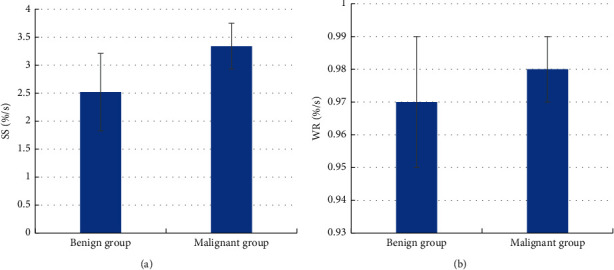
Comparison of parameters for the time-signal curve of MRI images of SPN patients. (a) Comparison of the SS value of MRI images of benign and malignant SPN patients; (b) comparison of WR values of MRI images of benign and malignant SPN patients. ^*∗∗*^indicates a remarkable difference between the benign and malignant SPN patients, *P* < 0.01.

In the time-signal curve, the two groups of SPN patients were compared for the SS and WR value of MRI images (Figure 9). The SS value in the benign group was (2.52 ± 0.69) %/s, and the SS value in the malignant group was (3.34 ± 00.41) %/s. Obviously, the SS value in the benign group was significantly lower than that in the malignant group (*P* < 0.01). The WR values of the two groups were not statistically different (*P* > 0.05).

### 3.7. Comparison of ADC Values of MRI Images at Different b Values of Benign and Malignant SPN Patients

**Figure 10:**
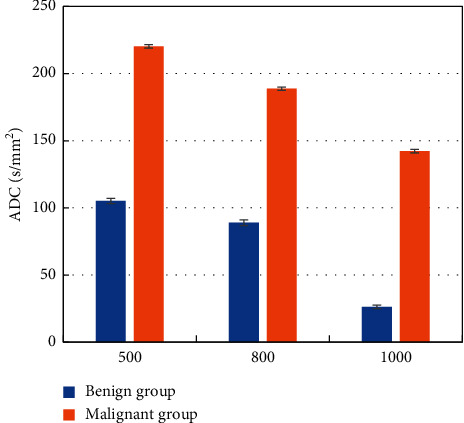
Comparison of ADC values of MRI images at different b values of benign and malignant SPN patients.^*∗∗*^indicates a remarkable difference between the benign and malignant SPN patients, *P* < 0.01.

At different *b* values, the two groups of SPN patients were compared for the ADC values of MRI images (Figure 10). At different *b* values, the ADC value of benign SPN patients was evidently lower than that of malignant group, showing a remarkable difference between the two (*P* < 0.01).

## 4. Discussion

The Dice index is also named as the F1 score in the information retrieval domain and has been widely applied in verification of the segmentation effect of 3D medical images [12]. The Dice index can be used to depict a better segmentation effect [13]. In this study, an MRI image segmentation model (SVM-L) was established based on the SVM algorithm, and its performance was evaluated factoring into DI, SE, SP, and MSE. The results herein showed that the DI, MSE, SE, and SP values of lung MRI images segmented through SVM-L models were obviously superior to those of MRI images segmented through other algorithms. These results manifested that the SVM-L model, which was constructed based on SVM algorithm, had better effect in segmentation of MRI images for the lung and was apparently superior to the nonoptimized SVM algorithms. As compared with the study results of Khan et al. [14], the SE and SP of lung MRI images segmented through the SVM-L model have been dramatically improved. The reasons are analyzed as follows: the well-trained SVM is used in the study to classify roughly segmented MRI images of the lung. The gray-level, gradient-level, texture characteristics of featured 2D MRI images as well as special information characteristics of 3D MRI images are entered to more precisely divide the lung position in MRI images and remove adhesion in the lung [15], so that SE and SP of MRI segmentation for the lung are notably raised, and its segmentation time is obviously shortened as compared to other algorithms.

At present, the multislice helical CT is the gold standard for assessing morphological characters of SPN and pulmonary distribution. However, the radiation dose of multitemporal scanning of dynamically enhanced CT quadruples is higher than that of the conventional CT scanning, the radiation hazard of which severely restricts its scope of application [16]. MRI is featured by multilayer, multisequence, and intensified signal characterizers. Its diagnosis rate of a pulmonary nodule with *d* > 5 mm attains 100% [17]. The study results herein manifested that, in terms of the proportion of patients in time-signal curve types of different MRI images, there was no statistical difference between the benign and malignant SPN patients (*P* > 0.05). It is very difficult to identify benign and malignant SPN using MRI curve types, which is consistent with the research results put forward by Feng et al. [18] and Wielpütz et al. [19]. The SS value in the time-signal curve of the MRI image is more relevant to the microvessel density of tumors [20]. The study results herein showed that the SS values of benign SPN patients were apparently lower than those of malignant patients (*P* < 0.01). According to analysis, such result was perhaps caused by active tumor vascular proliferation and a sharp increase in microvessel density inside the malignant SPN [21]. The WR in the time-signal curve of the MRI image is relevant to interstitial diseases (resilience and collagenous fiber) [22]. The study result herein showed that there was no statistical difference in WR values in the time-signal curve of MRI images of the two groups of patients (*P* > 0.05), which was consistent to the research result of Guan et al. [23]. As the *b* value is growing, the diffusion-weight of the MRI image is also increasing, and the ADC value is closer to the actual diffusion value of the tissue; meanwhile, the MRI image encounters a drop in the signal-noise ratio and deformation under the interference of a magnetic susceptibility artifact of the lung [24]. The result herein manifested that when the *b* value was within 500–1000, the ADC value of MRI images of benign SPN patients was apparently lower than that of the malignant group (*P* < 0.01). The optimal *b* value and ADC threshold to distinguish benign and malignant SPN with MRI should be selected from a larger number of samples for further discussion.

## 5. Conclusions

In this article, the SVM-L model was established based on SVM algorithm and was applied to process MRI images for the lung, to differentiate benign and malignant SPNs. The result showed that the SVM-L model highly raised the segmentation effects of MRI images for the lung, which was suggested in clinic. However, the study still has many shortcomings; for example, the sample size is small, which will reduce the power of the study. An expanded sample size is necessary to confirm the optimal *b* value and ADC threshold to differentiate benign and malignant SPNs using the SVM-L model. In conclusion, the SVM-L model can significantly improve the segmentation effects of MRI images and increase the accuracy to differentiate benign and malignant SPNs, providing reference for the diagnosis and treatment of SPN patients.

## Data Availability

No data were used to support this study.
